# Supplementary material for: Mutations in PCYT2 disrupt etherlipid biosynthesis and cause a complex hereditary spastic paraplegia
Source: Brain. 2019 Oct 22;142(11):3382–97. doi: 10.1093/brain/awz291 (PMC6821184; doi:10.1093/brain/awz291)
Supplement: awz291_Supplementary_Data [file awz291_supplementary_data.zip › awz291-Suppl_data/Supplementary_Data4.zip!/Supplementary information 4 - plasma lipidomics.zip!/Plasma lipidomics/Control vs Patient2/Statistical plots and data/PCA.pdf]

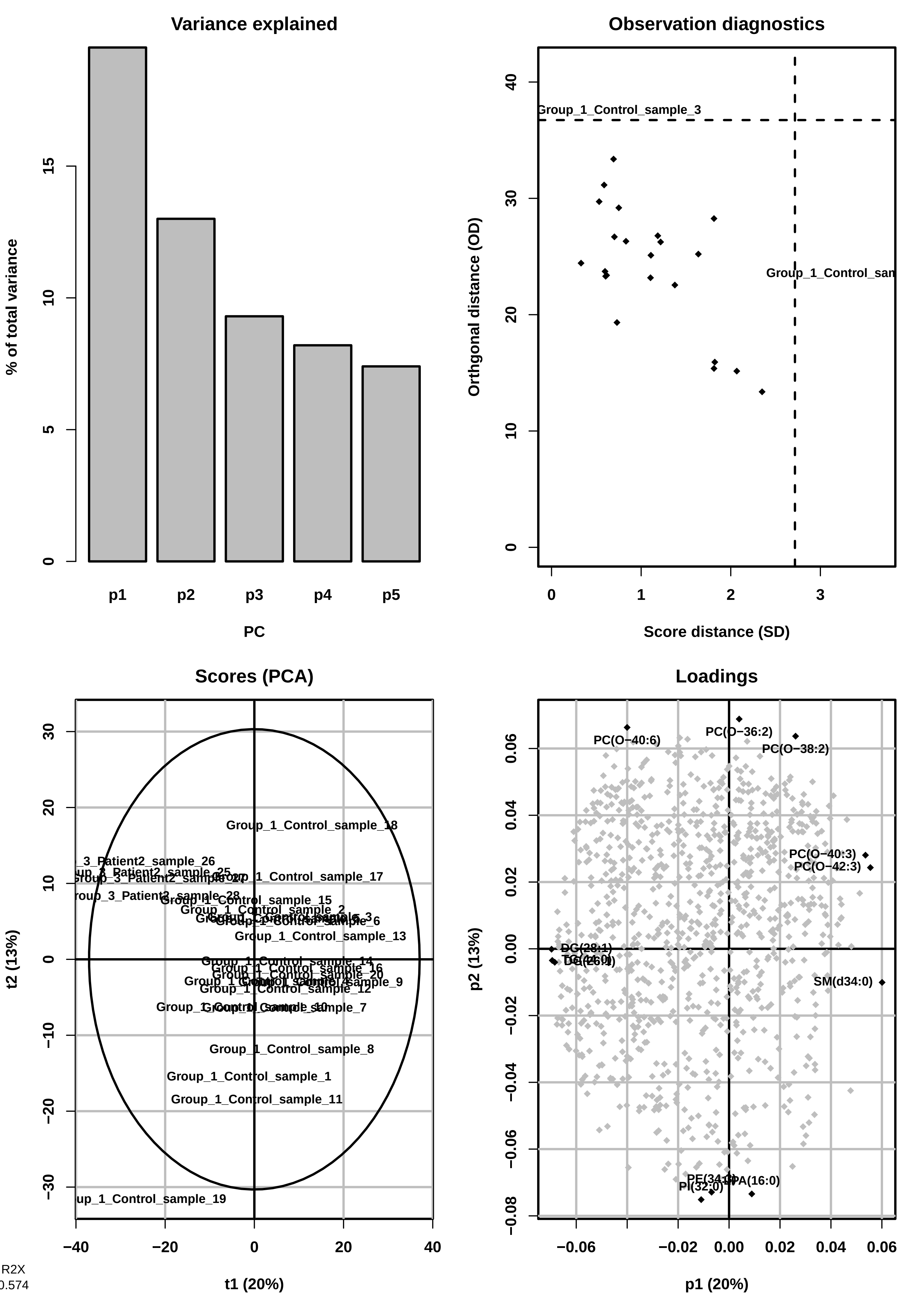

Variance explained
% of total variance
0
5
10
15
p1
p2
p3
p4
p5
PC
Observation diagnostics
Orthgonal distance (OD)
Score distance (SD)
Group_1_Control_sample_3
Group_1_Control_sam
Scores (PCA)
t2 (13%)
t1 (20%)
Group_1_Control_sample_18
Group_1_Control_sample_17
Group_1_Control_sample_15
Group_1_Control_sample_13
Group_1_Control_sample_6
Group_1_Control_sample_12
Group_1_Control_sample_7
Group_1_Control_sample_8
Group_1_Control_sample_1
Group_1_Control_sample_11
Patient2_sample_26
Group_3_Patient2_sample_28
Group_3_Patient2_sample_27
up_1_Control_sample_19
Loadings
p2 (13%)
p1 (20%)
PC(O−40:6)
PC(O−36:2)
PC(O−38:2)
PC(O−40:3)
PC(O−42:3)
DG(28:1)
SM(d34:0)
PI(32:0)
PA(16:0)
R2X
0.574
